# Mark Twain's Story of His Life

**Published:** 1907-10

**Authors:** 


### MARK TWAIN'S STORY OF HIS LIFE.

Mark Twain refuses to let his captivating autobiography be published in book form until after his death, but journalistic enterprise has come to the rescue, and we are to have Mark's masterpiece after all. He has consented to let it appear as a serial. It has been secured at enormous cost by the Sunday Magazine of the Chicago Record-Herald, which has a name for capturing big prizes of this sort, such as Conan Doyle's "Sir Nigel" and Kipling's "Sons of Martha."

Thus it falls out that the readers of The Sunday Record-Herald are to have a delightful treat without extra cost. For months to come Mark Twain will go on telling in his droll way about the famous people he has met, how he came to create Colonel Sellers and Tom Sawyer, and all the funny things that have happened to him. The whole is to be profusely illustrated. The first installment—in the issue of October 27—is accompanied by a magnificent portrait of the humorist. Everybody who likes Mark Twain will want to read this great biography.

